# Supplementary material for: The impact of generative artificial intelligence on socioeconomic inequalities and policy making
Source: PNAS Nexus. 2024 Jun 11;3(6):pgae191. doi: 10.1093/pnasnexus/pgae191 (PMC11165650; doi:10.1093/pnasnexus/pgae191)
Supplement: pgae191_Supplementary_Data [file pgae191_supplementary_data.docx]

**
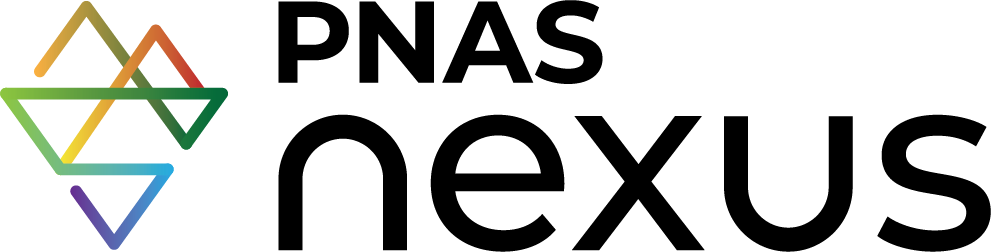
**

**Supplementary Information for**

The impact of generative artificial intelligence on socioeconomic issues and policy making.

Valerio Capraro, Austin Lentsch, Daron Acemoglu, Selin Akgun, Aisel Akhmedova, Ennio Bilancini, Jean-François Bonnefon, Pablo Brañas-Garza, Luigi Butera, Karen M. Douglas, Jim A.C. Everett, Gerd Gigerenzer, Christine Greenhow, Daniel A. Hashimoto, Julianne Holt-Lunstad, Jolanda Jetten, Simon Johnson, Werner H. Kunz, Chiara Longoni, Pete Lunn, Simone Natale, Stefanie Paluch, Iyad Rahwan, Neil Selwyn, Vivek Singh, Siddharth Suri, Jennifer Sutcliffe, Joe Tomlinson, Sander van der Linden, Paul A. M. Van Lange, Friederike Wall, Jay J. Van Bavel, Riccardo Viale

Paste corresponding author name here

Email: [valerio.capraro@unimib.it](mailto:valerio.capraro@unimib.it)

**This PDF file includes:**

Supplementary text

Tables S1 to S4

SI References

| **Future research directions** | | | |
| --- | --- | --- | --- |
| **Research area** | **Specific question** | **Potential design** | **Trade-off** |
| Investigate how AI can be used to make information more accessible, especially for individuals with disabilities. | Can AI-based summarization tools improve information accessibility for individuals with cognitive disabilities by simplifying complex texts? | Evaluate the comprehension of complex news articles by individuals with cognitive disabilities after using AI-based summarization tools, compared with individuals not using these tools. | Summarization could make content more accessible, but oversimplification might omit critical details, challenging the balance between accessibility and content accuracy. |
|  | **How can AI-generated audio descriptions for visual media impact comprehension and enjoyment for visually impaired users?** | Compare the experience of visually impaired users consuming visual media with and without AI-generated audio descriptions, measuring both comprehension and enjoyment levels. | While AI-generated audio descriptions can significantly enhance the accessibility and enjoyment of visual media, overly detailed descriptions could distract or confuse. |
|  | **Can AI-powered mobility assistants improve navigation in public spaces for individuals with physical disabilities?** | Evaluate the impact of AI-powered assistants on the independence and mobility of individuals with physical disabilities in public settings. | AI-powered assistants may increase independence and accessibility to public spaces; but over-reliance on AI assistants might inhibit the development of personal navigation skills. |
| Understand how the largest firms could monopolize the future of AI; find ways for smaller and innovative firms to effectively compete with those largest players. | Does open-source AI decrease the risk of monopolization by large firms? | Compare the growth and success rates of firms that contribute to or use open-source AI versus those relying on proprietary solutions. | Open-source could democratize AI development, but the capacity of small firms to benefit from it may be smaller compared to large firms’, affecting the potential of such measures to reduce the competitive gap. |
|  | Which regulatory frameworks can effectively counteract the anticompetitive advantages gained through data monopolies? | Evaluate the effectiveness of various regulatory frameworks (e.g., data sharing mandates) in promoting competition. | Regulation could prevent monopolistic behaviors, but overly stringent regulations might stifle growth and innovation, particularly for smaller firms navigating compliance complexities. |
|  | How does access to venture capital affect the ability of small AI firms to innovate and compete? | Analyze the correlation between venture capital funding levels for small firms and their subsequent innovation output and market performance. | Venture capital could provide crucial resources for innovation, but the focus on rapid returns might push firms toward short-term gains over sustainable innovation. |
| Explore regulatory measures to prevent misuse or inappropriate access to data by AI systems. | Can a standardized transparency protocol prevent data misuse and privacy breaches? | Develop a standardized transparency protocol and test its impact on the frequency of data misuse and privacy breaches. Measure also users’ trust in the system and data sharing. | **Increased transparency may decrease data misuse and privacy breaches, but the difficulty of transparency protocols may actually decrease public trust and data sharing.** |
|  | What is the effect of data anonymization techniques on data misuse and personalization of information? | **Test various data anonymization techniques and measure their impacts on data misuse, privacy breaches, and personalization of AI-filtered information.** | **Data anonymization techniques can reduce data misuse and privacy breaches, but may also reduce the personalization and usefulness of AI-filtered information.** |
|  | How do opt-in versus opt-out data consent models affect user willingness to share data with AI systems for personalized content filtering? | Experiment with opt-in and opt-out consent models, measuring changes in user data sharing behavior and the effectiveness of personalized content filtering. | Opt-in models might increase user trust and consent quality, but result in less data being shared, possibly reducing the personalization effectiveness of AI systems. |
| Investigate strategies to identify and limit the spread of misinformation generated by AI. | Can dialoguing with gen AI reduce beliefs in misinformation? | Compare misinformation beliefs between participants engaging with an AI-system designed to counter misinformation and those interacting with a neutral AI. | AI's capabilities for personalization, linguistic fluency, and logical reasoning can diminish misinformation beliefs, but the risk of hallucinations could generate new misinformation. |
|  | Can machine learning models trained on AI-generated text effectively distinguish between human and AI-generated misinformation? | Train machine learning models on datasets containing human and AI-generated texts, testing their ability to accurately classify unseen texts. | Unique linguistic fingerprints left by AI might enable effective differentiation, but AI’s ability to mimic human writing styles challenges detection efforts with potential negative consequences in case of misclassification of misinformation. |
|  | Can verification systems on social media and messaging platforms reduce the spread of bot-generated misinformation? | Introduce a user verification system to distinguish between human and bot accounts on messaging platforms, analyzing the impact on misinformation spread in one-to-one communications. | Verification could reduce bot-driven misinformation, but the very existence of verification systems may decrease trust in the platform. |
| Explore ways to design AI-systems that support cooperative and ethical behavior in human-machine interactions. | **Do humans become more unethical when they can delegate decisions to AI compared to other humans?** | Subjects make morally ambiguous decisions, with the option to delegate these decisions either to AI or to another human, observing changes in their ethical standards. | The emotional detachment of AI may reduce moral responsibility, but the potential for AI to document and expose decisions might increase personal accountability. |
|  | What features of AI systems and users’ understanding of them promote human-AI cooperation? | Compare the extent of human cooperation with AI-systems in social dilemma situations, experimentally manipulating relevant interface design features and system descriptions. | Well-designed AI systems may improve cooperation, but cooperation may not reach the level observed in human-human interaction. |
|  | **How do different cultural perceptions of ethics influence the design of AI systems for global human-machine interaction?** | Design AI systems with ethical guidelines informed by diverse cultural norms. Test these systems in various cultural contexts, assessing acceptance and effectiveness in promoting ethical interactions. | Cultural customization could increase the global applicability and acceptance of AI systems, but reconciling conflicting ethical norms poses significant challenges for universal AI design. |
| Examine how AI-enhanced search engines can be designed to preserve user autonomy and plurality of information. | **Does making the data sources of AI-enhanced search engines transparent influence user engagement with alternative information sources?** | Disclose the data sources for AI algorithms in search engines and assess whether users seek out additional, alternative sources of information as a result. | Transparency about data sources might encourage users to seek diverse information, but could also lead to information overload. |
|  | **How do interactive AI assistants that guide users in refining search queries influence the range of information explored?** | Deploy interactive AI assistants that help users refine their queries and track the breadth of information they explore compared to users without assistance. | Interactive guidance could expand the scope of information users consider, but reliance on suggestions might limit independent exploration. |
|  | **What role does user education on search engine algorithms play in preserving information plurality?** | Implement an educational program about how AI search algorithms work and measure its impact on users’ ability to seek out diverse information sources. | Educating users could enhance their ability to find diverse information, but overemphasizing algorithmic literacy may burden users without addressing underlying biases in the algorithms. |
| Consider how the proliferation of AI-generated content could lower the quality of online information and ensure that human users can continue to contribute new knowledge. | **How does the ratio of AI-generated to human-generated content affect content diversity on online forums?** | Manipulate the ratio of AI-generated to human-generated content in an online forum environment. Survey users on perceived content diversity and quality. | Higher ratios of AI content could enhance content availability, but may also homogenize the content or lead to mediocre or over-creation of banal, “average” content. |
|  | **How does the ratio of AI-generated to human-generated content influence human users’ motivation to contribute original content?** | Manipulate the ratio of AI-generated to human-generated content and measure the extent to which humans contribute original content over time. | A higher proportion of AI content could provide users with more information, potentially increasing creativity, but also potentially saturating the platform and demotivating human contributors. |
|  | **Can AI content curation algorithms be trained to prioritize and highlight innovative human-generated content over derivative AI-generated pieces?** | Develop an AI curation algorithm that differentiates between innovative human-generated content and derivative AI content. Measure its impact on the visibility of human contributions. | Prioritizing human creativity enhances content diversity and innovation, but algorithmic bias towards human-generated content could ignore valuable AI contributions or insights. |
| \| Investigate the role of Corporate Digital Responsibility and its implementation challenges \|  \| \|  \| \|  \| \| --- \| --- \| --- \| --- \| --- \| --- \| \|  \| \|  \| \|  \| \| \|  \| \|  \| \|  \| \| | What are the main barriers to implementing effective digital responsibility strategies in organizations? | Development of organizational strategies to implement a general Corporate Digital Responsibility framework. | Many organizations might not be aware of the role and importance of corporate digital responsibility strategies, which might cause over-regulation. |
|  | How does the cost-benefit analysis of digital responsibility practices influence organizational commitment? | Develop predictive models to forecast the long-term benefits and costs associated with digital responsibility practices. | Stakeholders may be wary of decisions made on the basis of analysis if the process is not transparent or if the rationale behind predictions and recommendations is not clearly understandable. |
|  | In what ways can regulation support or hinder the adoption of good corporate digital responsibility practices? | Engage in comparative policy analysis and legal research to understand the landscape of existing regulations affecting corporate digital responsibility practices across different jurisdictions. | While regulatory analysis can provide a detailed understanding of the legal framework, it might not fully capture the operational and strategic challenges faced by organizations in complying with these regulations. |

*Table S1. Summary of the main research directions on the impact of generative AI on information. For each relatively broad research area, we propose three specific research questions, along with potential experimental designs. We also identify an underlying theoretical trade-off that complicates the derivation of a priori hypotheses. This list is not exhaustive but rather provides examples of the kinds of questions future research should aim to address.*

| **Future research directions** | | | |
| --- | --- | --- | --- |
| **Research area** | **Specific question** | **Potential design** | **Trade-off** |
| Investigate how AI can be designed and implemented to augment human skills and increase productivity, rather than to simply replace workers and forego the long-run productivity upsides of maximizing workers’ contributions to production. | How do organizations balance automating knowledge tasks versus hiring more knowledge workers for efficiency and innovation? | Analyze organizational decisions, assessing the ratio of investment in automation technologies to the recruitment of knowledge workers. | Automation offers cost efficiency and 24/7 productivity, but the unique insights brought by human knowledge workers present a compelling case for augmentation over full automation. |
|  | What is the role of AI in enhancing the creative process for designers and artists without diminishing originality? | Artists and designers use AI tools in a controlled study, with assessments of creativity, efficiency, and originality, compared to a control group not using AI. | Balancing AI’s input with human creativity involves complex interactions that could affect the originality of outputs unpredictably. Additionally, bias in training data (e.g., Western oriented) might diminish creativity. |
|  | Does the use of generative AI free up human resources for more strategic tasks within organizations? | Organizations implement generative AI tools in a pilot program and track the allocation of human resources to strategic tasks versus before AI implementation. | The assumption that AI frees up human resources could be challenged by the need for increased oversight and quality control. |
| Examine how AI can facilitate more access to economic opportunities, particularly through reducing language-related barriers and promoting remote work technologies that can democratize access to the digital economy. | What role does AI play in enabling marginalized groups, including countries in the Global South, to access remote work opportunities in the digital economy? | Analyze employment trends and outcomes in marginalized groups before and after introducing AI-enabled remote work platforms. | AI could provide anonymized job matching, enhancing access, but restrictions on technology use could negate these benefits. |
|  | Does generative AI reduce language barriers in cross-cultural collaboration, especially with regard to low-resource language? | Organizations introduce AI-driven language translation tools in some teams but not others, measuring collaboration effectiveness and project outcomes. | AI improves communication but might limit personal language growth and cultural insight. For low-resource languages, scant training data can increase translation errors. |
|  | Can AI-powered job matching platforms more effectively connect remote workers with global employment opportunities compared to traditional platforms? | Compare employment success rates and job satisfaction between remote workers using AI-powered job matching platforms and those using traditional platforms. | AI’s ability to match skills with opportunities may improve job market efficiency, but biases in AI algorithms and digital divide could perpetuate inequalities. |
| Conduct long-term studies to monitor the evolving impact of AI on the workforce, capturing both the immediate and delayed effects on work across educational and occupational strata. | How does continuous use of AI tools impact job satisfaction and employee burnout? | Track job satisfaction and burnout rates among employees using AI tools versus those using traditional methods over multiple years. | AI tools might boost innovation and creativity, but could also lead to higher expectations and workload, decreasing satisfaction and increasing burnout. |
|  | Can AI-driven educational tools reduce the skill gap between workers from different educational backgrounds? | Implement AI-based educational tools and measure skill levels before and after intervention across workers with varying backgrounds. | AI personalized learning could upskill workers, but differences in digital literacy and access may widen the skill gap. |
|  | What are the long-term effects of AI on work-life balance across different occupational sectors? | Longitudinal survey assessing work-life balance pre- and post-AI adoption in varying sectors. | AI might offer more flexible working conditions, but increased monitoring and job demands could negatively impact work-life balance. |
| Explore how AI can be utilized in educational and training programs to encourage basic competency with generative AI tools and better-equip workers in vulnerable job sectors in anticipation of labor market changes. | How does intergenerational collaboration within teams, specifically focused to the exchange of AI tool knowledge, impact team performance, inclusivity perceptions, and psychological well-being? | Create diverse teams to assess the impact of intergenerational AI knowledge-sharing on performance, inclusivity perception, and well-being. | Intergenerational knowledge sharing might improve team performance, inclusivity perceptions, and well-being, but it could also introduce challenges such as resistance to change from senior members or frustration among juniors, with negative effects on the outcome variables. |
|  | Can AI-based personalized learning platforms increase the effectiveness of re-skilling programs for workers facing automation? | Workers facing job displacement are enrolled in re-skilling programs using AI-based personalized learning, compared to traditional re-skilling approaches. | Personalization may accelerate skill acquisition, yet differences in learning styles and technology familiarity among workers can produce a re-skilling gap. |
|  | How effective is generative AI in bridging the digital literacy gap for older workers participating in workforce re-entry programs? | Older workers in re-entry programs use generative AI tools aimed at improving digital literacy, comparing outcomes to those in programs without AI support. | AI tools could provide personalized, pace-adjustable learning, but pre-existing technology apprehensions among older workers may hinder engagement and effectiveness. |
| Research labor laws, taxation policies, and social support systems that could support workers displaced or disadvantaged by AI. | How do worker retraining and job replacement programs impact the economic stability of workers displaced by AI? | Incorporate AI-skills into pilot worker retraining programs in regions with high rates of AI-driven displacement, comparing against control groups in similar regions with alternative, non-AI worker retraining. | AI-centric worker retraining could enable workers to be more agile in finding re-employment in the event of job displacement, but this may not be successful for all workers, particularly those later in their careers or with fewer pre-existing technical skills. |
|  | How do changes in labor laws to protect workers influence their job security and income stability in AI-intensive industries? | Analyze workers’ job security and income before and after implementing new labor protections in AI-heavy sectors, comparing with sectors where laws remain unchanged. | Enhanced labor protections could improve job security and income, but increased operational costs for employers might reduce the availability of work. |
|  | What are the effects of AI on wage inequality within sectors, and can progressive taxation of AI-generated profits mitigate these effects? | Analyze wage distributions within sectors before and after the introduction of progressive taxation on AI profits, using control sectors without such taxation for comparison. | Progressive taxation might redistribute wealth and reduce inequality, but could also discourage AI investment and innovation, affecting sector growth and wage levels. |

*Table S2. Summary of the main research directions on the impact of generative AI in workplace environments, along with specific example questions and corresponding experimental design and theoretical trade-offs.*

| **Future research directions** | | | |
| --- | --- | --- | --- |
| **Research area** | **Specific question** | **Potential design** | **Trade-off** |
| Examine how generative AI can be effectively used for personalized learning. | How does the use of generative AI for personalized feedback in essay writing impact students’ writing proficiency over a semester? | Compare students in classes receiving personalized AI feedback with those receiving standard teacher feedback. Assess improvements in writing proficiency. | Personalized AI feedback could offer more tailored and immediate improvements, but may lack the psychological understanding and motivational impact of human feedback. |
|  | How do generative AI-driven simulations impact students’ understanding of abstract scientific concepts? | Science classes incorporate generative AI-driven simulations to teach abstract concepts, comparing students’ conceptual understanding and engagement to classes using traditional teaching methods. | Simulations could visually and interactively convey complex concepts. However, over-reliance on simulations might limit abstract understanding. |
|  | What is the long-term effect of using generative AI for homework assistance on students’ independent learning skills? | Students using generative AI tools for homework assistance are tracked over an academic year, comparing their development of independent learning skills to peers who do not use AI assistance. | AI tools could provide personalized help and boost learning efficiency, but might also reduce students’ initiative to tackle challenges and limit the development of independent learning skills. |
| Investigate how curricula can be redesigned to include generative AI as a tool for enhancing learning while also teaching students to critically engage with and understand this technology. | How does integrating generative AI in science curricula affect students’ understanding of complex concepts? | Implement generative AI in a subset of science classes, comparing students’ performance on concept understanding to those in classes without AI tools. | Generative AI may enhance concept understanding through personalized learning, but the potential for over-reliance on AI could hinder independent critical thinking skills. |
|  | Can generative AI tools assist in teaching problem-solving skills more effectively than conventional tools? | Compare the effectiveness of generative AI tools versus conventional tools in teaching problem-solving. | The adaptive learning algorithms of AI could offer customized problem-solving practice, but the potential for hallucinating reasonable but wrong mathematical solutions could hinder learning. |
|  | How does the use of generative AI for creating interactive history lessons affect students’ historical empathy? | Measure changes in historical empathy in students engaging with interactive, AI-generated history lessons versus traditional textbook-based learning. | Interactive content could deepen engagement and understanding, but overemphasizing technology might detract from human-based discussion and reduce historical empathy. |
| Study effective training methods for teachers to integrate AI tools into their teaching practices and identify the additional support required to manage these technologies in the classroom. | Can peer mentoring programs increase teachers’ confidence in using generative AI tools? | Implement a peer mentoring program where AI-experienced teachers mentor those less familiar, and assess changes in confidence and usage rates of AI tools in teaching. | Peer mentoring could provide useful support and encouragement, but mismatches in teaching styles might limit the program’s effectiveness and even reduce confidence. |
|  | How does collaborative training in AI tool integration affect teachers’ classroom implementation success compared to individual training methods? | Split teachers into groups receiving either collaborative or individual training on AI tool integration, followed by evaluations of their success rate of AI tool implementation in their classrooms. | Collaborative training could enhance teamwork and collective problem-solving skills, but individual differences in learning pace and style might lead to uneven skill acquisition and implementation success. |
|  | Can just-in-time training methods enhance teachers’ ability to integrate AI tools into their curriculum? | Implement a just-in-time training program for a group of teachers, and compare their integration success to teachers who received traditional training sessions. | Just-in-time training might offer immediately applicable knowledge, but the lack of comprehensive training could leave teachers unprepared for broader integration challenges. |
| Explore strategies to ensure that use of generative AI leads to a diversity of educational experiences and outcomes | How does the educational use of generative AI affect students’ creativity? | Study three groups of students: one using generative AI with educator support for creativity, one using AI independently, and the other not using AI. Assess the creativity of their outputs. | Generative AI can expand students’ creativity with proper guidance but might reduce originality and insight if used without educational support. |
|  | How does the educational use of generative AI support access to a plurality of knowledge, viewpoints, and perspectives? | Test the effect of pedagogically advanced uses of generative AI on alternate viewpoints, divergent and critical thinking. | Generative AI can offer a broad spectrum of ideas and stimulate critical thinking with the right pedagogical approach, it risks reinforcing limited perspectives inherent in its training data if not carefully managed. |
|  | How does the educational use of generative AI support the inclusion of learners who are otherwise marginalized and disadvantaged? | Classrooms adopt AI tools and practices designed for inclusivity, measuring the academic and social integration outcomes of otherwise disadvantaged students compared to classrooms without such tools. | AI tools could enhance accessibility and learning for socially disadvantaged students and those with disabilities, but implementation challenges and the need for teacher training might even decrease integration outcomes. |
| Evaluate the long-term impacts of generative AI on student learning, teacher workloads, and educational outcomes. | Does the use of generative AI in lesson planning reduce teachers’ preparation time? | Track lesson preparation time for teachers using generative AI tools versus traditional planning methods over a semester. | AI tools could streamline the lesson planning process, but the initial learning curve and adjustments to AI suggestion might offset time savings. |
|  | How does long-term use of generative AI affect students’ critical thinking skills? | Compare critical thinking skill development over a school year between students taught with generative AI and those with a traditional curriculum. | Generative AI could provide more engaging and diverse content, potentially enhancing critical thinking, but might also lead to overreliance. |
|  | How does AI-based personalized learning impact long-term student motivation and academic persistence? | Track student motivation and persistence in academic programs using generative AI personalization versus traditional programs, over several years. | Personalized learning could boost motivation and persistence by aligning with students’ interests and needs, but potential overreliance might diminish intrinsic motivation. |

*Table S3. Summary of the main research directions on the impact of generative AI on education, along with specific research questions, with corresponding experimental design and theoretical trade-offs.*

| **Future research directions** | | | |
| --- | --- | --- | --- |
| **Research area** | **Specific question** | **Potential design** | **Trade-off** |
| Research how AI can assist healthcare professionals in diagnosis, treatment planning, and patient monitoring. | Does AI-assisted medical decision making improve patient outcomes including decision accuracy and bias reduction? | Healthcare providers are given the option to consult or not consult medical advice from generative AI chatbots, with decision accuracy and decision bias measured across medical contexts and healthcare provider characteristics. | Clinicians could heed the advice of AI, improving their medical decision making. However, mistrust for AI tools, low AI literacy, or other sources of suboptimal interactions with AI could render AI-assisted decision making ineffective or detrimental for patient outcomes. |
|  | How does AI-assisted diagnosis compare to traditional diagnostic methods in terms of diagnosis accuracy? | Professionals diagnose using traditional methods in one group and AI assistance in another, comparing diagnostic accuracy. | AI could enhance diagnostic accuracy, due to its ability to analyze patterns in data, but over-reliance might overcome the clinician’s experiential intuition, potentially affecting diagnosis accuracy. |
|  | How does the integration of AI in patient monitoring impact the management of post-operative recovery? | Post-operative patients are monitored using AI-based systems in one group and traditional monitoring methods in another, comparing recovery rates, complications, and readmission rates. | AI monitoring could provide real-time data and early warning signs of complications, enhancing recovery management. However, reliance on technology might reduce direct patient-clinician interactions, potentially impacting care quality. |
| Investigate the use of AI to reduce the administrative burden on healthcare providers through efficient electronic health records (EHR) management. | Does the integration of AI into EHR systems reduce the time healthcare providers spend on documentation compared to traditional EHR systems? | Healthcare providers are divided into two groups, one using AI-integrated EHR systems and the other using traditional EHR systems, with the time spent on documentation measured over a specified period. | AI could streamline documentation processes, reducing time spent on paperwork. However, learning curves associated with new AI systems might initially increase the time required for documentation. |
|  | Can AI-powered speech-to-text solutions enhance the accuracy of clinical documentation in EHRs compared to manual typing by healthcare providers? | Providers use AI-powered speech-to-text for clinical documentation in one group, while another group relies on manual typing. The accuracy and completeness of records are compared. | Speech-to-text could improve efficiency and accuracy by reducing typing errors. However, speech recognition errors could introduce inaccuracies, affecting record quality. |
|  | **Can AI in EHRs reduce healthcare costs associated with administrative tasks?** | Analyze financial records to compare administrative costs before and after AI implementation in EHR systems. Include both direct costs and indirect costs. | AI could lower long-term costs by increasing efficiency, but the upfront costs of AI technology and necessary training could be substantial. |
| Study how AI can contribute to the development of personalized medicine, adapting treatments to individual patient needs and reducing healthcare disparities. | Can AI-based predictive models accurately identify patients at high risk for diabetes (or any other disease) and guide early intervention strategies? | Develop AI models using patient health data to predict diabetes risk and apply intervention strategies based on model predictions. Compare the incidence of diabetes in this group with a control group receiving standard care. | Predictive models could allow for earlier and more targeted interventions. However, inaccuracies in predictions could lead to unnecessary interventions or miss at-risk individuals. |
|  | How effective is AI in optimizing vaccine distribution strategies to maximize coverage and equity in diverse populations? | Use AI to design vaccine distribution strategies in diverse populations, comparing coverage and equity to strategies developed through traditional methods. | AI could optimize distribution logistics, enhancing coverage and equity. However, algorithmic biases might inadvertently exacerbating disparities. |
|  | Can AI-enhanced remote patient monitoring systems reduce hospital readmission rates? | Compare readmission rates of patients monitored using AI-enhanced systems with those of patients monitored using standard remote systems. | Enhanced monitoring might detect early signs of deterioration, reducing readmissions. Yet, overreliance might delay seeking in-person care when needed, potentially risking patient health. |
| Investigate strategies to increase public trust and understanding of AI in healthcare. | Does providing patients with detailed explanations of AI diagnostic processes improve their trust in AI-driven healthcare? | Patients receiving AI-driven diagnostics are split into two groups, one receiving detailed explanations and the other receiving standard information. Trust levels are measured post-interaction. | Detailed explanations could demystify AI processes, increasing trust. However, overly technical or complex information might overwhelm patients, potentially reducing trust. |
|  | What role does personalized communication from AI healthcare systems play in patient satisfaction and trust? | Implement AI healthcare systems that use personalized communication with patients, comparing patient satisfaction and trust to systems using generic communication. | Personalized communication might foster a sense of care and understanding. Yet, if perceived as insincere, it could diminish trust and satisfaction. |
|  | How does media campaign depicting AI’s role in healthcare influence public perception and trust? | Analyze public perception and trust in AI before and after targeted media campaigns depicting AI’s role in healthcare, comparing with a control group not exposed to the campaigns. | Positive media representation could improve public perception and trust in AI, but sensationalism or negative portrayals in some media could exacerbate fears and distrust. |
| Research how AI can improve healthcare accessibility in underserved regions and populations, in both rural and urban areas. | Can AI-driven mobile health applications effectively increase healthcare access in rural communities? | Implement an AI-driven mobile health application in rural communities. Compare health outcomes, access to care, and self-management behaviors with a control group not using the application. | Mobile health apps could significantly improve access and self-management, but poor internet connectivity and low digital literacy in rural areas might limit effectiveness and even widen disparities. |
|  | Can AI algorithms improve the accuracy of disease outbreak predictions in underserved areas, leading to better preparedness and response? | Develop AI algorithms to predict disease outbreaks in underserved areas, comparing the accuracy of predictions and subsequent response efforts with historical outbreaks managed without AI predictions. | Improved prediction accuracy could significantly enhance outbreak response. Yet, reliance on incomplete or biased data sets might result in inaccurate predictions, potentially misguiding response efforts. |
|  | How effective are AI-based systems in detecting early signs of mental health issues in underserved communities? | Use AI-based systems to monitor and detect early signs of mental health issues in underserved communities, compared with traditional detection methods. | AI might offer early and accurate detection, but misinterpretations or privacy concerns could hinder the trust and effectiveness of these systems. |
| Investigate the potential of AI to facilitate social connections, particularly for individuals with difficulties in forming relationships, while also studying the potential risks of over-reliance on AI for social interaction. | Can AI-based chatbots effectively reduce feelings of loneliness compared to traditional social programs? | Participants are divided into two groups, with one interacting with AI-based chatbots and the other engaging in traditional social programs. Measures of loneliness and social skills are assessed over time and their influence on health. | AI agents could offer constant companionship, potentially reducing loneliness. However, overreliance on AI could inhibit motivation for human interaction leading to increased isolation. Further, the lack of genuine human interaction might not fulfill deep social needs, affecting overall health. |
|  | How can AI be used to increase the quality of digital social interaction and communication? | Compare the human-to-human digital exchanges (emails, texts, DM, etc) when AI pro-social prompts are offered to those without prompts. | AI could prompt people to be more respectful, kind and courteous. The con is that people may view such messages as less authentic. |
|  | How does reliance on AI for social interaction impact the development of social skills in adolescents? | Track social skill development in adolescents who heavily rely on AI for social interaction versus those who primarily engage in human interactions, assessing communication skills, empathy, and relationship quality. | AI interaction might offer continuous social practice, but the absence of complex human feedback from multiple social companions could flatten social skills. |

*Table S4. Summary of the main research directions on the impact of generative AI on healthcare, along with specific example questions.*
